# Expertise Related Changes in Resting‐State Functional Connectivity Patterns Following a Clinical Reasoning and Decision‐Making Task

**DOI:** 10.1002/brb3.71153

**Published:** 2026-01-13

**Authors:** Filomeno Cortese, Pamela Hruska, Kevin J. McLaughlin, Sylvain P. Coderre, Andrea B. Protzner, Olave E. Krigolson, Kent G. Hecker

**Affiliations:** ^1^ Department of Community Health Sciences, Cumming School of Medicine University of Calgary Calgary AB Canada; ^2^ Hotchkiss Brain Institute University of Calgary Calgary AB Canada; ^3^ Research Department Interior Health Authority Kelowna BC Canada; ^4^ Department of Medicine University of Calgary Calgary AB Canada; ^5^ Department of Psychology University of Calgary Calgary AB Canada; ^6^ The Mathison Centre For Mental Health Research & Education University of Calgary Calgary AB Canada; ^7^ The Theoretical and Applied Neuroscience Laboratory University of Victoria Victoria BC Canada; ^8^ Faculty of Veterinary Medicine University of Calgary Calgary AB Canada

**Keywords:** clinical reasoning, expertise, functional magnetic resonance imaging, resting‐state, univariate and multivariate analyses

## Abstract

**Purpose:**

This study investigated the behavioral and resting‐state neural correlates of clinical decision‐making among expert gastroenterologists and novice medical students, aiming to understand how diagnostic expertise is reflected in either pre‐task and/or post‐task brain activity.

**Method:**

Participants completed a clinical decision‐making task while behavioral measures (accuracy and response time) were recorded. Resting‐state fMRI data were acquired immediately before and following the task. Group differences in brain connectivity were analyzed using seed‐based connectivity and multivariate partial least squares (PLS) analyses, focusing on the frontopolar prefrontal cortex (FPPFC) and its associated networks.

**Finding:**

Experts outperformed novices in diagnostic accuracy and speed, especially on “easy” cases, suggesting enhanced cognitive efficiency. Experts also showed more pronounced response time variation with task difficulty, potentially reflecting strategic modulation. Resting‐state fMRI revealed that experts had increased post‐task connectivity between the FPPFC and the paracingulate gyrus (PaCG), a brain area associated with the executive control network. Novices, by contrast, showed stronger FPPFC connectivity with the posterior cingulate cortex (PCC), part of the default mode network (DMN), indicating a return to internally directed cognition. PLS analyses further revealed that experts engaged executive and attentional network regions post‐task, while novices primarily activated DMN regions. Notably, for the expert group only, increased brain activity in attention‐related regions was associated with gastroenterologists who had slower, deliberate responses on easy cases.

**Conclusion:**

Clinical expertise is associated with sustained engagement of goal‐directed neural networks after task completion, potentially reflecting ongoing cognitive evaluation or preparation. In contrast, novices appear to disengage more readily, reverting to self‐referential thought. These findings highlight distinct neural mechanisms that may support the development of diagnostic expertise.

## Graphical abstract

Post‐task resting‐state fMRI revealed different network states for expert gastroenterologists and novice medical students. Experts showed increased prefrontal cortex (PFC)–paracingulate connectivity (executive control) suggesting sustained, goal–directed engagement after decisions. Whereas novices showed stronger PFC–posterior cingulate cortex connectivity (default mode) indicating they reverted back to internally directed cognition.

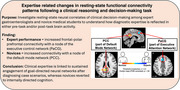

## 1 Introduction

Experts in a particular domain develop specialized cognitive and perceptual abilities through years of dedicated learning, training, and practice (Ericsson and Krampe 1993; Ericsson 2004). Experts can arrive at a more accurate diagnosis with increased efficiency through heightened use of non‐analytical, intuition‐based reasoning processes (Eva 2005; Norman and Brooks 1997). We define expert performance as reproducible superior performance on representative tasks, driven by deliberate practice and refined cognitive representations rather than by seniority alone (Ericsson and Krampe 1993; Ericsson and Smith 1991). This yields faster reasoning, more accurate decisions, and heightened perceptual sensitivity as compared to novices (Carrigan et al. 2019; Coderre et al. 2003; McLaughlin et al. 2007). In medicine, for instance, expert diagnosticians can rapidly identify anomalies in imaging or clinical patterns that novices often miss (Norman 2005). These expressions of expert performance provide behavioral benchmarks for interpreting neural differences.

The acquisition of such expertise is not only performance‐based but also reflected in structural and functional changes in the brain. Neuroimaging studies have demonstrated that prolonged domain‐specific training induces neuroplastic adaptations, including changes in both gray matter volume and activation patterns (Chang 2014; Gauthier et al. 2000; Hervais‐Adelman et al. 2015; Popescu et al. 2019; Woollett et al. 2009). For example, taxi drivers with extensive navigational experience show increased posterior hippocampal volume, a region implicated in spatial cognition (Maguire et al. 2003). Similarly, expertise in sports and music is associated with heightened activation in motor and auditory association cortices, respectively, compared to novices (Calmels 2020; Herholz and Zatorre 2012; Margulis et al. 2009).

Understanding the neural underpinnings of expertise may provide a basis for identifying training strategies most likely to enhance performance. Furthermore, this knowledge may clarify why some individuals improve at different rates or reach higher levels of achievement. Research utilizing functional magnetic resonance imaging (fMRI) in medical education has primarily focused on tasks that involve decision‐making (Downar et al. 2011; Durning et al. 2014; Melo et al. 2011) or visuospatial (Bahrami et al. 2011; Haller and Radue 2005) processes. However, only a limited number of studies have investigated clinical reasoning processes that are not visually based. One such study examined the neural correlates of analytical versus non‐analytical reasoning during medical diagnosis (Durning et al. 2012) in board‐certified experts in internal medicine. Participants underwent fMRI scanning across three distinct phases: reading (baseline), answering diagnostic questions, and reflecting on their reasoning. Following the scan, a think‐aloud protocol was employed to capture participants’ cognitive strategies. Greater activation in the prefrontal cortex (PFC) was interpreted as being associated with analytical reasoning, inferred from instances involving incorrect responses, guessing, and deeper cognitive engagement. A subsequent paper using these data employed a diagnostic thinking inventory after the fMRI session to determine whether self‐reported reasoning styles correlated with specific neural activation patterns (Durning et al. 2016). Results showed individual differences in inventory scores that reflect memory structure were associated with individual differences in activity in brain regions implicated in non‐analytical reasoning, including the left inferior parietal lobule, left ventromedial prefrontal cortex, and left dorsolateral prefrontal cortex (DLPFC). In contrast, individual differences in scores that reflect flexibility in thinking were linked to activity differences in regions associated with analytical reasoning, such as the bilateral ventromedial PFC and the right parahippocampal gyrus. These differentiated contributions of PFC subregions have been identified and supported in a recent systematic review and meta‐analysis (Cera et al. 2025) focused specifically on expert medical performance.

These previous fMRI studies examined the neural correlates of clinical reasoning in medical professionals and thus focused exclusively on experts. While these studies provided valuable insights into the neural systems engaged during expert‐level clinical decision‐making, they did not include a comparative novice group, limiting inferences about how such neural processes evolve over the course of training. In contrast, two sets of studies (Durning et al. 2015; Hruska et al. 2016a; 2016b; 2017a; 2017b) directly addressed this gap by comparing brain activation patterns between expert clinicians and medical students during clinical reasoning tasks. Two of these comparison studies targeted gastroenterology, a specialty that has otherwise received relatively little neuroimaging attention. Durning et al. (2015) investigated non‐analytic (i.e., non‐declarative) reasoning in a cohort of internal medicine interns (novices) and board‐certified internists (experts). The experimental task consisted of three phases: an initial reading phase in which a multiple‐choice question was presented, a decision phase in which response options were displayed for selection, and a final reflection phase during which participants were instructed to silently consider the reasoning behind their chosen diagnosis. Findings from this study indicated that both novices and experts engaged a shared neural network during non‐analytical reasoning. However, within this network, experts exhibited greater neural efficiency (Neubauer and Fink 2009) with reduced activation in the PFC. This decreased prefrontal activity was interpreted as evidence of more proficient use of non‐analytical reasoning strategies among experts, consistent with dual‐process theories of reasoning (Evans and Stanovich 2013). Hruska et al. (2016a; 2016b; 2017a; 2017b) investigated the neural differences between second‐year medical students (novices) and practicing gastroenterologists (experts) as they read and reasoned through sixteen clinical cases of varying difficulty in an MR scanner. Both groups showed activation in occipital, prefrontal, parietal, and temporal regions during the reading phase of the task (Hruska et al. 2016b; 2017b). However, novices exhibited significantly greater activation in the PFC, particularly during complex cases, suggesting a heavier reliance on working memory resources. In contrast, experts demonstrated more efficient neural processing, indicative of streamlined cognitive strategies developed through experience. During the decision‐making phase of clinical reasoning, both groups activated similar brain regions when diagnosing easy, straightforward cases; however, significant differences emerged while evaluating complex cases (Hruska et al. 2016a; 2017a). Novices showed increased activation in regions associated with factual, rule‐based processing, including the left anterior temporal cortex and left ventrolateral PFC. Experts, however, exhibited greater activation in regions linked to experiential knowledge and evaluative judgment, including the right dorsolateral and ventrolateral PFC and right parietal cortex. These behavioral and brain findings are consistent with the idea that expertise in clinical decision‐making involves a shift from analytical, rule‐based reasoning to more intuitive, experience‐based processing.

It is intuitive to investigate expertise‐associated differences in brain activity patterns that are related to a specific task, that is, clinical decision‐making, while participants (e.g., an expert or a novice) perform a clinical reasoning task. The studies mentioned above presented clinical cases to participants while measuring their brain activity with the blood‐oxygen‐level‐dependent (BOLD) signal (Ogawa et al. 1990). The investigators identified group differences in neural responses associated with clinical reasoning by using a general linear model to map activation patterns (Friston et al. 1994). This methodology, task‐based fMRI, has been used in identifying localized activation related to different types of memory (Cabeza et al. 2002; Cohen et al. 1997; Buckner et al. 1996), language (Binder et al. 1997), and motor activity (Bandettini et al. 1992). Task‐based fMRI remains the gold standard for delineating the functionally specific brain patterns associated with cognitive operations. Resting‐state fMRI (rs‐fMRI) is another useful paradigm that uses a task‐free design to identify brain areas that are functionally connected and may show differences across groups.

The human brain is a dynamic system, continually fluctuating in an organized manner even in the absence of overt task engagement. Resting‐state fMRI measures spontaneous, low‐frequency BOLD signal fluctuations while participants are not engaged in externally driven tasks. These spontaneous fluctuations, first described by Biswal et al. (1995), form spatially distinct, temporally synchronized networks, referred to as resting‐state functional connectivity networks, that correspond closely to task‐evoked activation patterns (Cordes et al. 2000; Toro et al. 2008; Smith et al. 2009). Canonical resting‐state networks include motor (Biswal et al. 1995), sensory (De Luca et al. 2005; Damoiseaux et al. 2006), cognitive control (Dosenbach et al. 2007; Vincent et al. 2008; Cole et al. 2010), memory (Vincent et al. 2008), attention (Fox et al. 2006), salience (Seeley et al. 2007), and default‐mode networks (Raichle et al. 2001; Greicius et al. 2003; Buckner et al. 2008). Resting‐state fMRI has proven especially valuable for studying clinical populations (Buckner et al. 2008; Greicius et al. 2004; Castellanos et al. 2008; van der Wijk et al. 2022), developmental trajectories (Damoiseaux et al. 2006), and individual differences in cognition (Mueller et al. 2013), given its task‐free nature and minimal demands on participants.

Methodologically, the key distinction is that task‐based fMRI captures condition‐dependent responses to stimuli, while rs‐fMRI reflects the ongoing, intrinsic, baseline organization of brain function. Resting‐state fMRI work often focuses on the default mode network (DMN), which is associated with directed cognition (Andrews‐Hanna et al. 2014), memory construction (Robin and Moscovitch 2017), mental simulation (Smallwood et al. 2021), and valuation (Clithero and Rangel 2014). The DMN's core hubs (medial prefrontal cortex, posterior cingulate/precuneus, and lateral temporal–parietal regions) interact dynamically with control and salience networks to balance internal interactions with external demands (Buckner et al. 2008; Spreng et al. 2010; Murphy et al. 2018). Conceptually, task fMRI indexes condition‐dependent responses, that is, what changes when a specific cognitive operation is engaged. Resting‐state fMRI, by contrast, indexes how large‐scale networks are organized when no task is imposed. These perspectives are complementary, meaning intrinsic architecture can constrain and predict an individual's task activations, and recent experience during a task can reconfigure resting connectivity over minutes to hours (e.g., consolidation or “replay” phenomena) (Smith et al. 2009; Tavor et al. 2016 Apr 8; Tambini and Davachi 2019).

For example, pre‐task resting‐state network function can influence how the brain responds to incoming stimuli and can be used to predict future neural responses, as well as behavioral and cognitive outcomes. Greicius and Menon (2004) observed that greater engagement of the DMN during rest was linked to greater engagement of task‐related regions during tasks. On the flip side, resting‐state connectivity is responsive to tasks performed immediately before the at‐rest scan session. Studies have shown that when participants engage in motor (Waites et al. 2005), memory (Albert et al. 2009; Tambini et al. 2010), and attentional (Stevens et al. 2010) tasks prior to resting‐state, spontaneous activity such as in the DMN, is altered due to these previous cognitive states. This task‐induced carryover effect on resting‐state functional connectivity has important implications for the effects of learning and experience. As such, resting‐state approaches provide a powerful framework for investigating individual differences in brain function, including those that may arise from accumulated expertise in domains such as medicine.

In the context of medical decision‐making, while the previously reported task‐based studies have shown experience‐related changes in brain activity (Durning et al. 2012; Durning et al. 2015; Hruska et al. 2016a; 2016b; 2017a; 2017b), it remains unclear whether functional differences are evident at rest among individuals with varying levels of clinical experience associated with performing a clinical decision‐making task. Here we present the first fMRI study that focuses on differences between medical novices and experts during resting‐state collected before and after performing a clinical reasoning task. The aim of this study was to examine differences in resting‐state networks between expert gastroenterologists and novice medical students. Specifically, we examined whether pre‐task resting‐state function differentiates experts from novices and if potential differences can be linked to performance on a subsequent task. We also examined how the intervening task modulated functional post‐task connectivity differences between these groups with a focus on regions that support accurate and efficient diagnostic reasoning.

## 2 Method

### 2.1 Participants

Twenty‐four right‐handed adults with normal or corrected‐to‐normal vision and no known neurological disorders provided informed consent. Two participants from the previously reported cohort (Hruska et al. 2016a; 2016b; 2017a; 2017b) did not complete the post‐task resting scan due to fatigue and neck pain and are therefore excluded from this study. Four additional datasets were excluded from the previous cohort due to technical problems; however, two of these were successfully recovered and replaced the two incomplete cases. The final sample for the current study included 20 participants with complete pre‐ and post‐task resting‐state scans. Our “novice” participants were ten second year medical students from the University of Calgary (8 male, mean (range) age 25.8 (22 to 38) years, SD = 4.6) and our “expert” group were ten practicing gastroenterologists (5 male, mean (range) age 39.6 (32 to 50Neubauer and Fink 2009; Evans and Stanovich 2013; Ogawa et al. 1990; Friston et al. 1994; Cabeza et al. 2002; Cohen et al. 1997; Buckner et al. 1996; Binder et al. 1997; Bandettini et al. 1992; Biswal et al. 1995) years, SD = 5.3). The experts all had formal academic teaching responsibilities at the University of Calgary, Alberta, Canada. This study was conducted in accordance with the ethical standards prescribed in the Declaration of Helsinki, the Calgary Health Ethics Research Board (CHREB), and the Seaman Family MR Research Centre.

### 2.2 Procedure

Six‐minute resting‐state scans were acquired before and after a clinical reasoning task, as acquired for Hruska et al. (2016a; 2016b), where participants read sixteen gastroenterology clinical scenarios. Eight scenarios were “easy” in that patient information was concordant with the analytical data presented in the scenario, and eight were “hard” in that the patient's data were discordant with the analytical data presented. For each scenario, participants were given 80 seconds to read a clinical case and then were asked to indicate the most likely diagnosis, presented in the form of a multiple‐choice question with four answer choices. Participants had 20 seconds to make their selection. Following their decision, they were provided feedback on the accuracy of their selection (i.e., the correct answer was highlighted on the multiple‐choice display). For more detail on the task, please refer to Hruska et al. (2016a; 2016b). During resting‐state scans, participants were asked to relax, blink normally, and let their mind wander (i.e., “not to think about anything in particular”) while looking at a white fixation cross on a grey background. The cross was projected (Avotec, Inc., Stuart, FL) on a screen which was positioned at the back of the MR scanner and viewed by the participant through a mirror mounted on the head coil above the eyes.

#### 2.2.1 Behavioral Data

Because neural measures derived from the resting‐state scans were analyzed for correlation with task performance, the behavioral data reported here overlap with those reported in Hruska et al. (2016a; 2016b). For each scenario, participants’ accuracy (% correct) and response time (ms) were recorded. Two‐way analysis of variance (ANOVA) was used to assess overall accuracy and mean response time between medical experience (novice, expert) and scenario difficulty (easy, hard). An ⍺‐level of 0.05 was assumed for these analyses.

#### 2.2.2 Functional and Structural Magnetic Resonance Imaging Data Acquisition

All images were collected using a 3‐Tesla GE MRI scanner (Discovery MR750, GE Healthcare, Waukesha, WI) equipped with a twelve‐channel phased‐array head coil at the University of Calgary, Seamen Family MR Research Centre at Foothills Medical Centre. Three‐dimensional high‐resolution (1 mm × 1 mm × 1 mm) T1‐weighted anatomical images were collected for anatomical registration of the fMRI data (TR/TE = 8.9/4.1 ms, matrix size = 384 × 256 × 112, field of view (FOV) = 25.6 cm × 25.6 cm). During the functional scans, both resting‐state and task‐based, T2*‐weighted gradient‐echo echo planar images (GRE‐EPI) were acquired with the following parameters: TR/TE = 2000/20 ms, flip angle = 70°, FOV = 24 cm × 24 cm, 64 × 64 matrix. Each functional run began with six TRs during which no data were acquired to allow for steady‐state tissue magnetization. A total of 180 echo‐planar imaging volumes were collected in each resting‐state functional run (one expert participant had 160 volumes collected post‐task due to fatigue); thirty‐seven 3 mm thick slices were collected.

#### 2.2.3 Functional MRI Data Preprocessing

Functional MRI data processing was carried out using FMRI Expert Analysis Tool (FEAT) Version 6.0, part of FSL (FMRIB's Software Library, www.fmrib.ox.ac.uk/fsl) (Jenkinson et al. 2012). The following preprocessing steps were applied: motion correction using the Motion Correction FMRIB's Linear Registration Tool (MCFLIRT (Jenkinson et al. 2002)); slice‐timing correction; non‐brain removal using the Brain Extraction Tool (BET (Smith 2002)); spatial smoothing using a Gaussian kernel with a Full Width at Half Maximum (FWHM) of 5.0 mm; and highpass temporal filtering (> 0.01 Hz). Linear registration of functional data to the high‐resolution structural and standard space images was carried out using FMRIB's Linear Registration Tool (FLIRT) (Jenkinson et al. 2002; Jenkinson and Smith 2001). Motion parameters that were extracted by MCFLIRT were included as nuisance regressors, and volumes that exceeded a displacement of 2 mm in any direction compared to a reference volume in the middle of a scan acquisition were identified and censored in the subject‐level General Linear Model (GLM; see next section). Following these preprocessing steps, each dataset was decomposed to its independent components (ICs) using FSL's Multivariate Exploratory Linear Optimized Decomposition into Independent Components (MELODIC) tool (Beckmann and Smith 2004). The resultant decomposition was a linear mixture of different processes, the spatial distributions of which are time‐invariant and statistically independent. We then used FIX (FMRIB's ICA‐based X‐noiseifier) (Salimi‐Khorshidi et al. 2014) to identify and remove ICs that corresponded to artifactual processes in the data (e.g., signals from white matter and cerebral spinal fluid parts of the brain).

#### 2.2.4 Seed‐based Functional Connectivity: Univariate Analysis

For each participant a resting‐state functional connectivity map (uncorrected voxel *p* threshold = 0.01) for each condition (pre‐ and post‐task) was calculated across the whole brain using the right frontopolar prefrontal cortex (FPPFC; MNI coordinates centered on 42, 54, 6) as the seed region of interest. The right FPPFC was selected based on the results of the clinical reasoning task‐based fMRI portion of this study (Hruska et al. 2016a; 2016b; 2017a; 2017b) that found both novice and expert clinicians showed similar and robust activation in this region during clinical reasoning processes. This region was also selected based on numerous neuroimaging and lesion studies that have implicated the FPPFC in high‐level cognitive functions such as decision‐making, prospective memory, and integration of multiple goals (Koechlin et al. 1999; Burgess et al. 2007; Boorman et al. 2009; Tsujimoto et al. 2011; Ramnani and Owen 2004; Mansouri et al. 2014). Based on these sources, the right FPPFC was a logical choice for the functional connectivity seed analysis.

For the first‐level analysis of each participant's resting state scan (pre‐task and post‐task), we computed seed‐based connectivity maps by entering the right FPPFC timeseries as a regressor of interest in a GLM with standard nuisance regressors (6 motion parameters; outlier volumes as single‐timepoint confounds). The resulting contrast of parameter estimates for *pre‐task* minus *post‐task* and *post‐task* minus *pre‐task* were *z‐*transformed, and the associated variance (i.e., uncertainty of the effect size at each voxel) images were retained. Within each participant, we modeled condition (post‐task vs. pre‐task) and computed the difference image, post‐task *minus* pre‐task, along with its propagated variance using FEAT fixed‐effects. This yielded one cope/varcope pair per participant representing the pre‐task to post‐task difference. Resultant difference images (cope/varcope) were forwarded to a group‐level mixed effects analysis using FMRIB's Local Analysis of Mixed Effects (FLAME1). We tested the group difference in condition:

$$\left({\text{Experts}}_{\mathrm{post}}-{\text{Experts}}_{\mathrm{pre}}\right)-\left({\text{Novice}}_{\mathrm{post}}-{\text{Novice}}_{\mathrm{pre}}\right)$$

The reverse contrast was also evaluated. Statistical maps were thresholded at *z* > 2.3 (voxel‐wise), and cluster significance was determined using a family‐wise error (FWE) correction of *p* < 0.05. We used the Montreal Neurological Institute (MNI) coordinates of the peak *z*‐statistic voxel within the cluster to determine the most probable anatomical cluster label from the Harvard‐Oxford Cortical Structural Atlas in FSL.

#### 2.2.5 Seed‐ and Behavior‐Partial Least Squares (PLS): Multivariate Analyses

Seed‐ and behavior‐PLS is a multivariate technique that enables the identification of the optimal brain signal patterns that differentiate conditions or groups in terms of functional connectivity and brain–behavior correlations (https://www.rotman‐baycrest.on.ca/index.php?section=84 (McIntosh et al. 1996), on the MATLAB R2016b platform, The MathWorks Inc., Natick, MA, USA). We first performed seed‐PLS to examine group (i.e., expert/novice) and condition (i.e., pre‐task/post‐task) dependent correlations between a seed region (i.e., right FPPFC) and the rest of the brain. This was followed by two behavior‐PLSs that examined group‐ and condition‐dependent correlations between behavior measures and the rest of the brain (McIntosh et al. 1996; Schreurs et al. 1997; McIntosh et al. 1998; Caplan et al. 2006) in pre‐task resting‐state and post‐task resting‐state conditions. These analyses allowed us to identify regions within each resting‐state condition that were correlated with clinical decision‐making performance (i.e., accuracy and response times for “easy” and “hard” case scenarios) as measured during the task‐fMRI in Hruska et al. (2016a; 2016b; 2017a; 2017b). The motivation of these multivariate analyses was to uncover functional networks that support better clinical reasoning skills due to experience. By comparing brain‐behavior relationships *before* and *after* a clinical reasoning decision‐making task, we examined whether or not resting‐state networks differed **between** medical experts and novices prior to a clinical decision‐making task and also mapped the influence of task performance on post‐task resting‐state. Any significant interactions were followed up by testing for simple effects.

For each analysis, singular value decomposition of the correlation matrix identified latent variables (LVs) that identified similarities or differences between (1) groups and conditions in the seed analysis and (2) groups and behavioral measures in the behavior analyses. For seed‐PLS, LVs indicated the degree to which each group and condition is related to the identified pattern of BOLD signal that is functionally connected to the right FPPFC. For the behavior‐PLS, LVs represented a common or differential brain‐behavior across groups.

Statistical assessment for the seed‐ and behavior‐PLSs was performed at two levels. First, the overall significance of each LV was assessed with 500 permutation tests (McIntosh et al. 2004; Good 2013). An LV was considered significant if the observed singular value exceeded the permuted singular value in more than 95% of the permutations (corresponding to *p* < 0.05). Second, bootstrap resampling, with replacement, with 500 samples was used to determine the consistency of the identified spatial pattern, or voxel saliences, within groups across participants. These standard error estimates required no corrections for multiple comparisons, as they were calculated in a single mathematical step on the whole brain. We designated a threshold of 2.1, corresponding roughly to a 95% confidence interval, or a *p*‐value of less than 0.05 (Efron and Tibshirani 1986).

## 3 Results

### 3.1 Behavior

**FIGURE 1:**
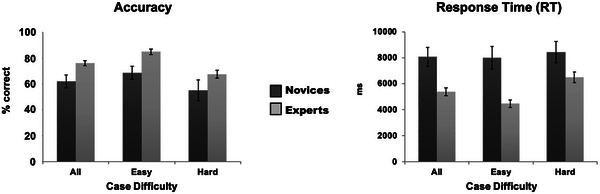
Group mean performance (left: accuracy; right: response times) and standard errors for Novices (dark gray) and Experts (light gray) during a clinical reasoning task while acquiring BOLD signal in an MR scanner (acquired during Hruska et al. (2016a; 2016b).

The two‐way ANOVA that examined the impact of expertise (novice vs. expert) and case difficulty (easy vs. hard) on diagnostic accuracy and response time identified three significant main effects (see Figure 1). First, expert gastroenterologists (77% mean ± 11% standard deviation) correctly diagnosed more cases than second‐year medical students (62% ± 22%), *F* (1, 36) = 7.99, *p* < 0.008. Second, the expert group (5484 ms ± 1517 ms) responded more quickly than the novice group (8229 ms ± 2608 ms), *F* (1, 36) = 17.93, *p* < 0.0002. Last, all participants were more accurate on easy (77% ± 15%) than on hard cases [(61% ± 20%), *F* (1, 36) = 9.36, *p* < 0.005]. There were no significant interaction effects.

### 3.2 rs‐fMRI: Univariate GLM

**FIGURE 2:**
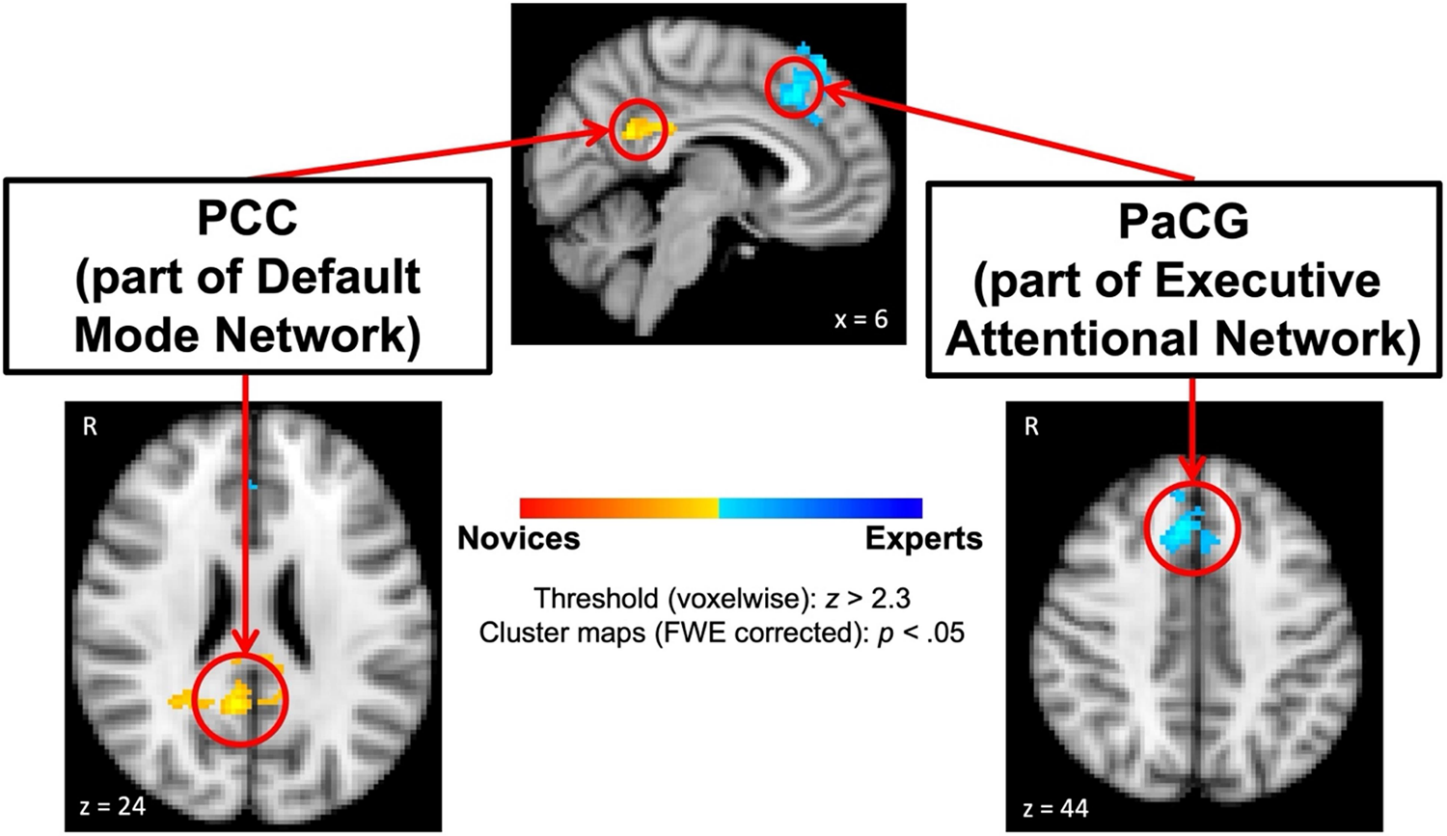
Univariate GLM group‐level mixed‐effects condition by group interaction in seed‐based connectivity. Maps show brain regions (red‐outlined circles) where post‐task > pre‐task rs‐fMRI between gastroenterologist experts (light/blue clusters) and novice second‐year medical students (yellow/orange clusters) overlaid on a standard MNI152 brain image. Abbreviations: FWE = family‐wise error, PaCG = paracingulate gyrus, PCC = posterior cingulate cortex, R = right hemisphere, x = sagittal slice, z = axial slice, *z* = *z*‐score threshold value used.

Group‐level mixed‐effects analyses revealed significant group (novices vs. experts) differences in changes in right FPPFC functional connectivity from pre‐ to post‐clinical decision‐making task. Specifically, experts showed a larger connectivity increase with the right paracingulate gyrus (PaCG; MNI152 standard space peak voxel coordinate of 6, 22, 44) compared to the novice group (see Figure 2, light‐blue/blue clusters). This brain region is a key node of the executive attentional network (Vincent et al. 2008; Seeley et al. 2007; Dosenbach et al. 2008; Power et al. 2011; Cole et al. 2014). In contrast, novices showed a larger connectivity increase with the right posterior cingulate cortex (PCC; MNI152 standard space peak voxel coordinate of 6, −47, 24) relative to the expert group (see Figure 2, yellow/orange clusters). This brain area is a core region of the default mode network (DMN; Raichle et al. 2001; Greicius et al. 2003; Buckner et al. 2008; Andrews‐Hanna et al. 2014; Fox et al. 2005).

### 3.3 rs‐fMRI Multivariate PLS

**FIGURE 3:**
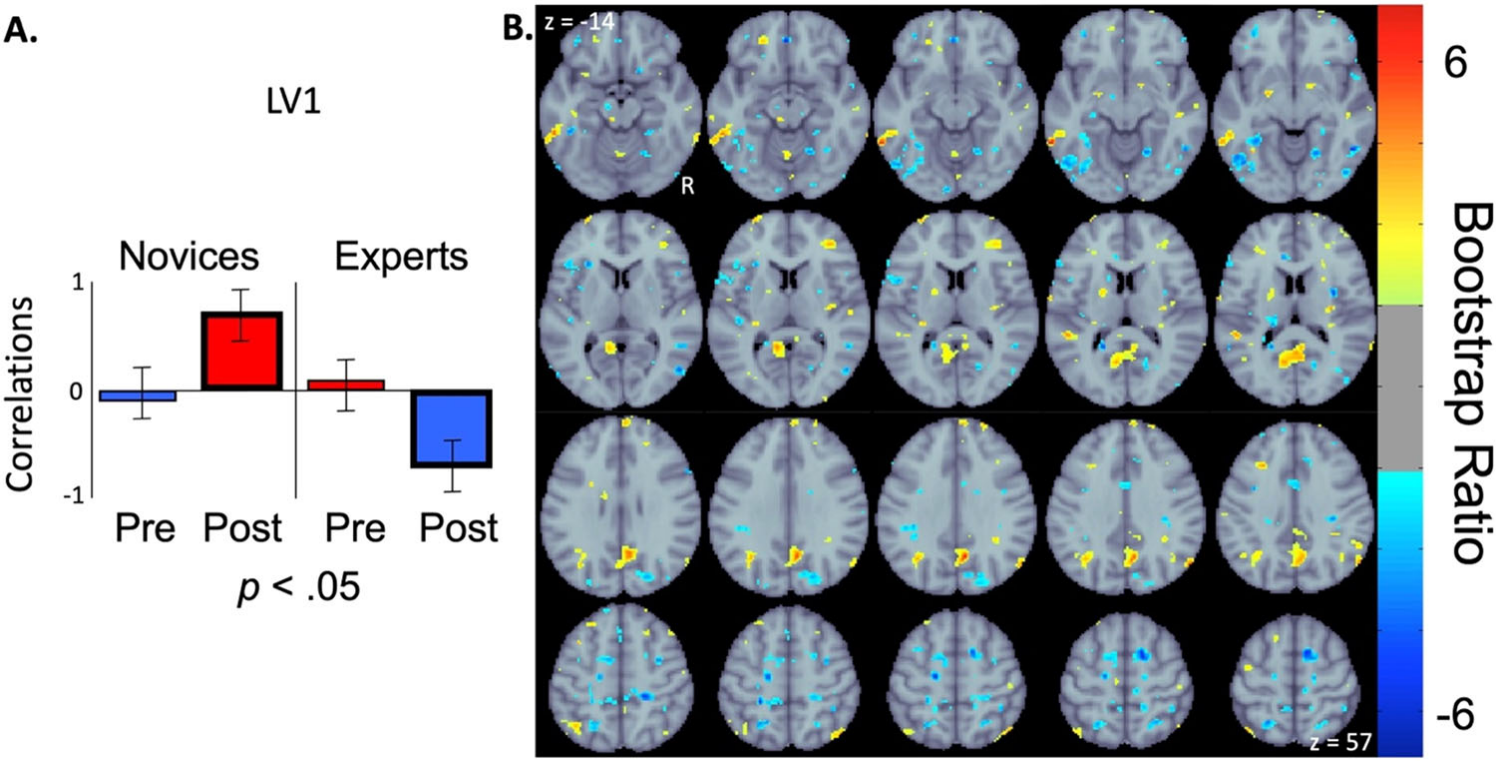
Correlations bar graph (A) and corresponding functional connectivity maps (B) resulting from seed‐PLS analysis examining the relationship between groups (Novices/Experts) and conditions (pre‐/post‐task resting‐state). The bar graph (A) indicates the correlations calculated from the first significant LV where group differences in post‐task resting‐state functional connectivity patterns with the seed region (right FPPFC) are identified in the spatial maps (B). Error bars indicate 95% confidence intervals from bootstrap estimation. Error bars that cross the *x*‐axis indicate unstable brain scores (i.e., not significant). The spatial maps (B) display yellow/red clusters representing brain regions with increased connectivity with the seed region in the novice group during post‐task resting‐state. The light‐blue/blue clusters represent brain regions with increased connectivity with the seed region in the expert group during post‐task resting‐state. Abbreviations: LV1 = first latent variable, Post = post‐task resting‐state condition, Pre = pre‐task resting‐state condition, R = right hemisphere, z = axial slice (MNI152 standard space).

The seed‐PLS analysis identified two significant LVs. The first LV (*p* < 0.05; 70.65% crossblock covariance explained) revealed both positive and negative correlations that differentiated between groups during the post‐task resting‐state condition. The pre‐task resting‐state condition did not contribute to this pattern, as their associated confidence intervals crossed the zero *x*‐axis. Brain regions with positive saliences (see Figure 3, yellow/red clusters) represent increased functional connectivity with the seed region post‐task for the novice group. This effect was strongest at the bilateral posterior cingulate cortex (PCC; MNI152 standard space peak voxel coordinates of 7, −55, 12 and −4, −53, 30), the right medial prefrontal cortex (mPFC; peak voxel coordinate of 26, 50, 8), the left frontal pole (FP; peak voxel coordinate of −20, 47, −14), the left middle temporal gyrus (MTG; peak voxel coordinate of −60, −43, −10), and bilateral angular gyrus (AG; peak voxel coordinates of 39, −50, 54 and −40, −64, 36). Brain regions with negative saliences (see Figure 3, light‐blue/blue clusters) represent increased functional connectivity with the seed region post‐task for the expert group. This effect was strongest at bilateral paracingulate gyrus (PaCG; peak voxel coordinates of 7, 18, 46 and −7, 16, 46), bilateral inferior temporal gyrus (ITG; peak voxel coordinates of 46, −35, −16 and −43, −54, −8), the left superior frontal gyrus (SFG; peak voxel coordinate of −26, −3, 50), and the right superior parietal lobule (SPL; peak voxel coordinate of 29, −48, 50).

**FIGURE 4:**
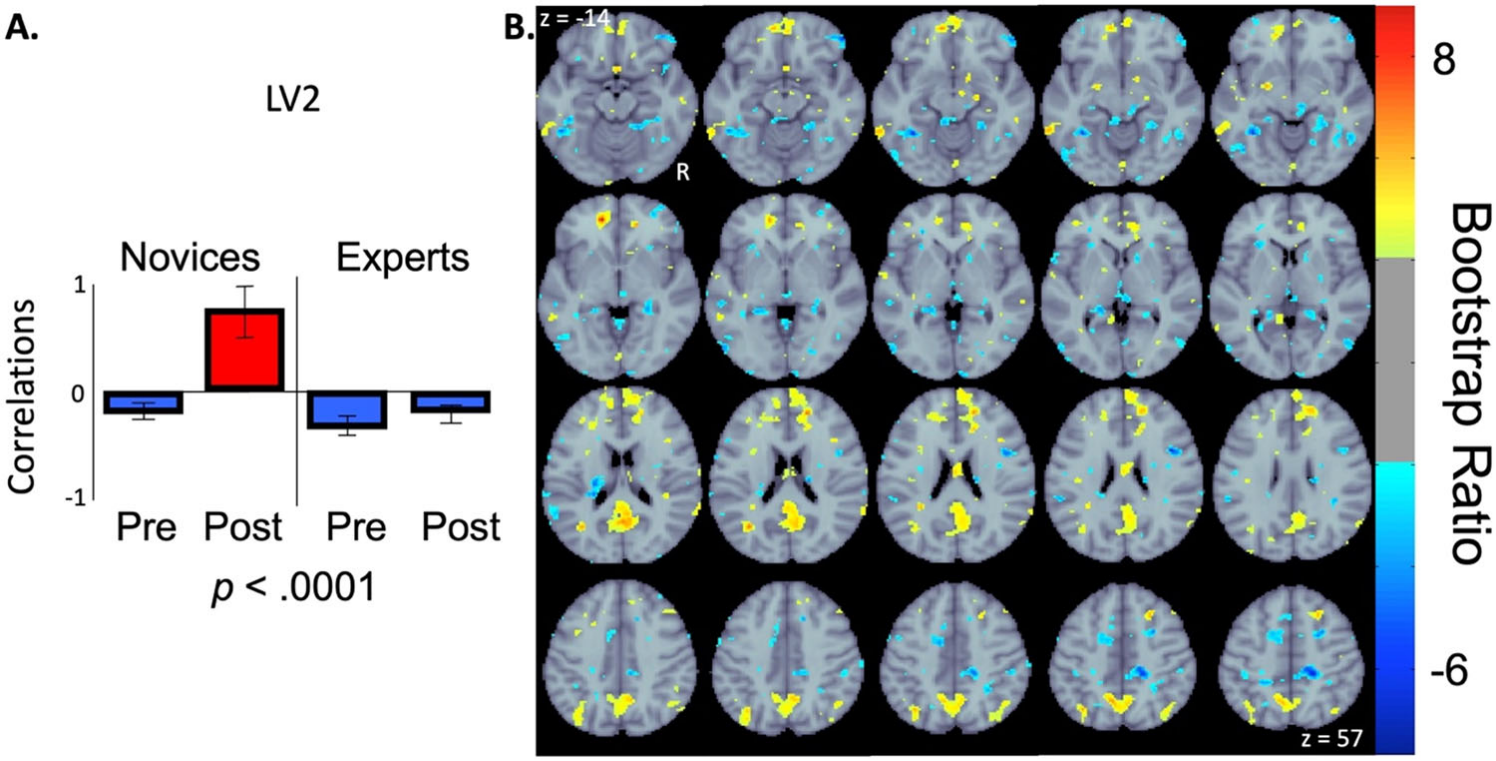
Correlations bar graph (A) and corresponding functional connectivity maps (B) resulting from seed‐PLS analysis examining the relationship between groups (Novices/Experts) and conditions (pre‐/post‐task resting‐state). The bar graph (A) indicates the correlations calculated from the second significant LV, where group differences in pre‐ and post‐task resting‐state functional connectivity patterns with the seed region (right FPPFC) are identified in the spatial maps (B). Error bars indicate 95% confidence intervals from bootstrap estimation. Error bars that cross the *x*‐axis indicate unstable brain scores (i.e., not significant). The spatial maps (B) display yellow/red clusters representing brain regions with increased functional connectivity with the seed region in the novice group during the post‐task resting‐state condition. The light‐blue/blue clusters represent brain regions with increased functional connectivity with the seed region in the novice group during the pre‐task resting‐state condition and the expert group in both pre‐ and post‐task resting‐state conditions. Abbreviations: LV2 = second latent variable, Post = post‐task resting‐state condition, Pre = pre‐task resting‐state condition, R = right hemisphere, z = axial slice (MNI152 standard space).

The second significant LV (*p* < 0.0001; 78.35% cross‐block covariance explained) revealed both positive and negative correlations that differentiated between the novice post‐task resting‐state condition and all other group/condition combinations. Brain regions with positive saliences (see Figure 4, yellow/red clusters) represent increased functional connectivity with the seed region for the novice group post‐task. This effect was strongest at bilateral posterior cingulate cortex (PCC; MNI152 standard space peak voxel coordinates of 5, −56, 18 and −7, −46, 32), bilateral medial prefrontal cortex (mPFC; peak voxel coordinate of ‐7, 54, ‐8), and bilateral angular gyrus (AG; peak voxel coordinates of 41, −61, 38 and −45, −57, 38). Brain regions with negative saliences (see Figure 4, light‐blue/blue clusters) represent increased functional connectivity with the seed region for both groups during the pre‐task resting‐state and for the expert group during the post‐task resting‐state. This effect was strongest at the left lingual gyrus (LG; peak voxel coordinates of −27, −46, −7), and the right precuneus cortex (peak voxel coordinate of 13, −42, 47).

**FIGURE 5:**
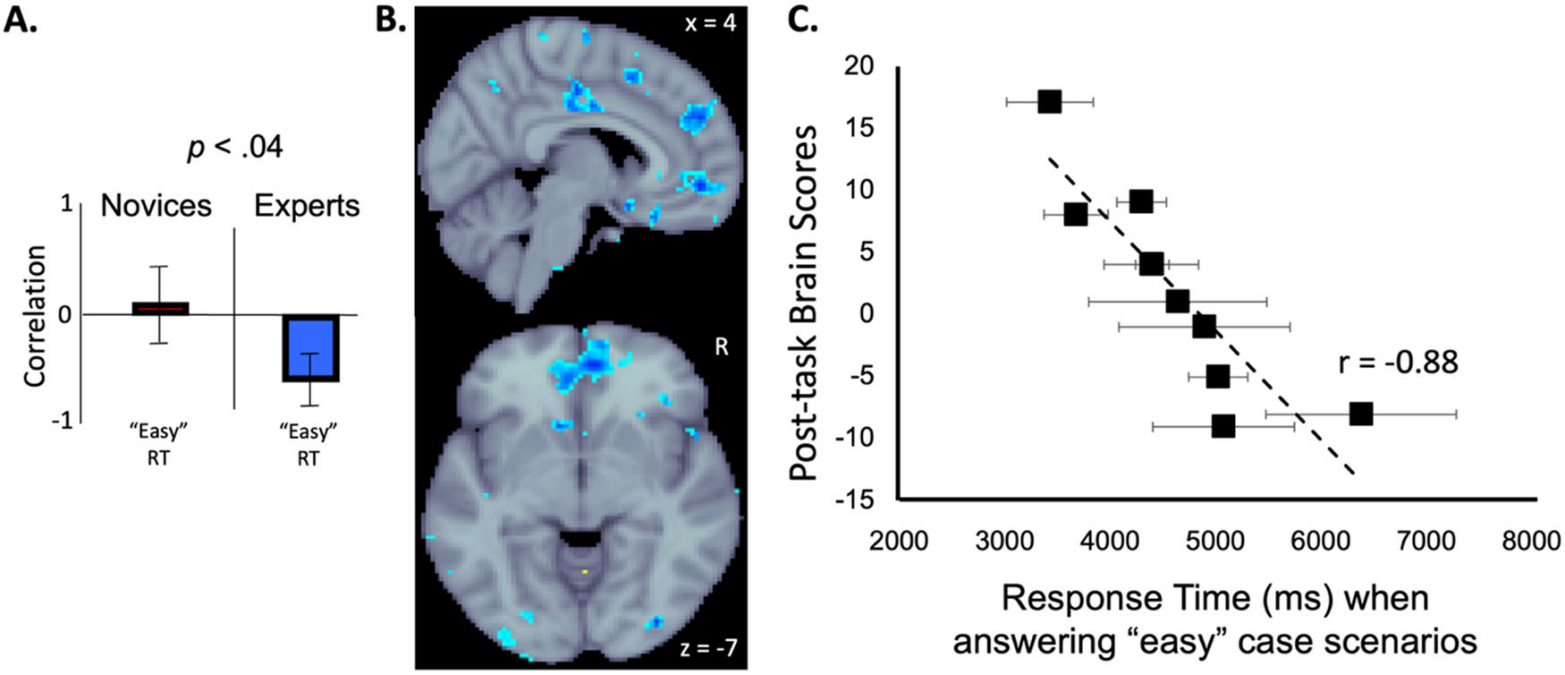
Correlations bar graph (A), brain activity maps (B), and scatterplot (C) describing the behavior‐PLS analysis examining the relationship between performance (i.e., response times in “easy” case scenarios between groups (Novices/Experts) and the post‐task resting‐state condition. The bar graph (A) indicates the group‐ and condition‐dependent correlations calculated from the only significant LV (*p* < 0.04) between response time performance and brain regions during the post‐task resting‐state condition identified in (B; light‐blue/blue clusters). Vertical error bars indicate 95% confidence intervals from bootstrap estimation. Error bars that cross the *x*‐axis indicate unstable brain scores (i.e., not significant). Scatterplot (C) shows the correlation between the post‐task resting‐state brain activity and response time performance while making clinical decisions for “easy” case scenarios. Each black square represents the average response time for each expert gastroenterologist when answering an “easy” case scenario. Horizontal bars represent the standard error of response times. Abbreviations: r = Pearson correlation coefficient of trendline, R = right hemisphere, x = sagittal slice, z = axial slice (both in MNI152 standard space).

The pre‐task behavior‐PLS analysis with the group (novices vs. experts) and the four behavioral measures (i.e., accuracy and response times for “easy” and “hard” cases) identified no significant effects. The post‐task behavior‐PLS analysis identified one significant LV (*p* < 0.05; 30.65% cross‐block covariance explained), reflecting an interaction effect. We then followed up with simple effects behavior‐PLS analyses for each behavioral measure. Only the analysis of response times for “easy” case scenarios yielded a significant LV (*p* < 0.04; 30.49% cross‐block covariance explained) showing a distributed pattern of brain regions (see Figure 5; light‐blue/blue clusters) where post‐task resting‐state activity correlated with task‐fMRI response times. The effect was strongest at bilateral paracingulate gyrus (PcCG; MNI152 standard space peak voxel coordinates of 6, 50, −7 and −7, 44, −7), the left superior frontal gyrus (SFG; peak voxel coordinate of −7, 33, 42), bilateral anterior cingulate gyrus (ACC; peak voxel coordinates of 2, −10, 38 and −2, −11, 34), and bilateral lateral occipital cortex (LOC; peak voxel coordinates of 29, −85, −6 and −37, −8, −6). A follow‐up Pearson correlation confirmed that more negative brain scores during post‐task resting‐state were associated with slower response times (*r* = −0.88).

## 4 Discussion

The present study investigated the behavioral and resting‐state neural correlates of clinical decision‐making among expert gastroenterologists and novice medical students. Behaviorally, experts demonstrated significantly higher diagnostic accuracy and faster response times than novices, consistent with previous findings that clinical expertise enhances efficiency and precision in diagnostic reasoning (Ericsson 2004; Norman 2005). These performance differences were also modulated by task difficulty, with both groups performing worse and slower on hard case scenarios. Difficulty had a notable impact on both accuracy and response time, suggesting that task complexity challenges cognitive resources irrespective of experience level.

Neuroimaging findings identified differences in how functional connectivity changed from pre‐ to post‐task performance between experts and novices. Experts showed increases in connectivity between the right FPPFC and the right PaCG, a region associated with executive attention (Vincent et al. 2008; Dosenbach et al. 2008; Ridderinkhof et al. 2004), indicating a shift toward task‐relevant, goal‐directed cognitive processing after decision‐making. This suggests that experts continue to engage top‐down attentional networks after task completion, possibly to consolidate decisions or evaluate task performance. In contrast, novices displayed stronger connectivity between the FPPFC and the PCC, a central hub of the DMN, suggesting a return to internally focused, self‐referential processing following the task (Raichle et al. 2001; Buckner et al. 2008; Andrews‐Hanna 2012). This may indicate that novices disengage from task‐relevant networks more readily, reverting to internally directed cognition.

Multivariate seed‐PLS analysis supported and further elaborated on group differences found with the univariate GLM analysis. The first LV indicated that post‐task resting‐state connectivity patterns diverged significantly between groups. It revealed that experts exhibited increased right FPPFC connectivity with regions linked to executive control such as the PaCG, ITG, and SFG during rest after the task, whereas novices showed stronger right FPPFC connectivity with DMN‐associated areas including the PCC, mPFC, and angular gyrus (AG) (Raichle et al. 2001; Andrews‐Hanna 2012; Fox and Raichle 2007). As a reminder, the right FPPFC was selected based on the results of the clinical reasoning task‐based fMRI portion of this study (Hruska et al. 2016a; 2016b; 2017a; 2017b) that found both novice and expert clinicians shared this region during clinical reasoning processes. These results suggest that task‐induced brain activity modulates subsequent resting‐state activity differently in experts versus novices. Expertise is associated with stronger connections with regions that support sustained attention and cognitive control even at rest, whereas novices show stronger connections with default mode regions after task completion.

The second latent variable further emphasized the post‐task resting‐state pattern characterized by increased functional connectivity with the classic DMN regions within the novice group, which distinguished them from both experts and pre‐task conditions. This pattern may reflect faster disengagement from task‐relevant cognitive processes in novices and is consistent with previous interpretations that novices revert to self‐referential thought after completing complex clinical tasks, potentially reflecting a lack of sustained task‐related mental framework (Spreng and Turner 2013).

Additionally, we identified a significant correlation between post‐task brain activity at rest and behavioral performance in the expert group. Increased brain activity in regions such as the ACC, PaCG, and LOC was correlated with slower response times on easy cases, indicating that slower decisions were associated with greater post‐task brain activity among these task‐relevant areas. These areas are well‐established components of networks supporting conflict monitoring, attention, and visual processing (Seeley et al. 2007; Dosenbach et al. 2008; Corbetta and Shulman 2002; Menon and Uddin 2010). This suggests a form of neural efficiency wherein experts are able to reinstate or maintain task‐relevant network states to facilitate performance (Pessoa 2014).

Contemporary models fractionate the DMN into at least three interacting subsystems: (i) a core (mPFC–PCC/precuneus) supporting self‐referential integration, (ii) a medial temporal (MT) subsystem (hippocampus, parahippocampus, posterior inferior parietal lobule (IPL)/AG) supporting episodic/semantic construction and scene building, and (iii) a dorsomedial subsystem (dmPFC, temporoparietal junction (TPJ), temporal poles) supporting mentalizing and abstract inference (Andrews‐Hanna et al. 2014; Smallwood et al. 2021; Andrews‐Hanna et al. 2010). Functions within these subsystems can be broadly characterized as “cold” (i.e., memory construction, semantic integration, perspective taking) and “hot” (valuation/affect‐laden appraisal concentrated in vmPFC and PCC) (Spreng et al. 2010; Bartra et al. 2013; Levy and Glimcher 2012). Reinterpreting our results through this perspective, the novice FPPFC–PCC coupling post‐task likely reflects greater engagement of the “cold” DMN core/MT subsystems. This is paired with concurrent involvement of mPFC/vmPFC and AG in our multivariate patterns engaging “hot” subsystems. By contrast, experts’ strengthened FPPFC–PaCG/SFG/ITG connectivity suggests sustained coupling of frontoparietal control with evaluative/attentional circuits and *reduced reliance* on the DMN immediately post‐task, relying predominately on “cold” subsystems.

Our findings emphasize that clinical expertise is not only reflected in overt task performance but also in the dynamic organization of large‐scale brain networks measured during resting‐state post‐task. Importantly, interpreting novice‐expert differences via DMN subsystems clarifies *which* networks are engaged after decision‐making (e.g., “cold” subsystems that generally support constructive memory (Robin and Moscovitch 2017; Smallwood et al. 2021; Benoit and Schacter 2015) or “hot” subsystems that are more closely associated with valuation (Clithero et al. 2014; Bartra et al. 2013; Levy and Glimcher 2014, 2012) and how they trade off with control networks. Increased connectivity among attentional and control networks after making clinical diagnoses may be a neural biomarker of expert performance, whereas increased connectivity among DMN core/MT subsystems post‐task may indicate a need for training that scaffolds the handoff from internal construction/appraisal back to goal‐directed maintenance (Mak et al. 2017). This study offers a complementary view to task‐based work of how neural engagement differs across levels of experience, highlighting functional distinctions that may underlie acquired diagnostic expertise.

## 5 Limitations

This study has several limitations. First, the study relied on a specialized group of medical school students and gastroenterologists with at least 10 years of clinical experience and therefore had a relatively small sample size. Small sample sizes can reduce the statistical power of the study and potentially fail to identify relevant functional connectivity similarities/differences between groups and reduce replicability. However, we used resampling statistics in PLS, which emphasizes reproducibility and reliability of the statistics rather than just null hypothesis testing. Bootstrap estimate confidence intervals ensure result stability, and multiple split‐half resamplings ensure reproducibility (Efron and Tibshirani 1986). Further studies with larger samples are encouraged to replicate and expand the current findings. Second, our expert group was on average 13 years older than our novice group. Expertise‐related changes in brain activity may present differently across age groups; for example, younger novices and older experts might rely on different strategies or neural networks when solving clinical problems (Reuter‐Lorenz and Park 2014; Kennedy et al. 2015). We, therefore, incorporated age difference as a nuisance variable into the study design to disentangle age‐related from expertise‐related neural changes. It was also encouraging that resting‐state functional connectivity network differences between groups only appeared after the clinical decision‐making task in both our univariate and multivariate analyses. Finally, for resting‐state fMRI, our scan length of 6 min is generally considered a good starting point. However, more recent work suggests that longer scan durations can improve the reliability of functional connectivity estimates, with some studies suggesting that scan durations of 10 to 15 min may be beneficial, especially for capturing group differences in connectivity (Ma et al. 2024). For future research we recommend using longer resting‐state durations to potentially obtain more stable functional connectivity maps.

## 6 Conclusions

Expert clinicians not only perform better behaviorally but also exhibit distinct patterns of task‐induced modulations in subsequent resting‐state connectivity that are linked to their superior performance. These differences underscore the importance of both task‐evoked and resting‐state brain dynamics in clinical reasoning studies. Future research could explore how training or feedback might foster more expert‐like brain patterns in novices and whether longitudinal changes in resting‐state connectivity can serve as biomarkers of developing expertise. These findings also have implications for medical education, particularly for designing interventions that go beyond skill acquisition to shape the neural underpinnings of clinical reasoning.

## Author Contributions

F. C., P. H., and K. G. H. designed the study. F. C. and P. H. collected data. F. C. and A. B. P. analyzed and interpreted the data. F. C. and K. G. H. prepared the draft plan. All authors contributed to writing the manuscript. All authors read and approved the final manuscript.

## Funding

This study was funded by the Cumming School of Medicine, University of Calgary.

## Ethics Statement

The research was approved by the University of Calgary Research Ethics Board (REB14‐1865). The researchers were informed about the study and written/verbal consent was obtained from all participants in accordance with the Declaration of Helsinki before data were collected. All methods were carried out in accordance with the relevant guidelines and regulations.

## Conflicts of Interest

The authors declare no conflicts of interest.

## Data Availability

The corresponding author upon reasonable request will provide data supporting the findings of this study.
